# Effect of the renin-angiotensin system on the exacerbation of adrenal glucocorticoid steroidogenesis in diabetic mice: Role of angiotensin-II type 2 receptor

**DOI:** 10.3389/fendo.2022.1040040

**Published:** 2022-11-17

**Authors:** Amanda da Silva Chaves, Nathalia Santos Magalhães, Daniella Bianchi Reis Insuela, Patrícia Machado Rodrigues E. Silva, Marco Aurélio Martins, Vinicius Frias Carvalho

**Affiliations:** ^1^ Laboratory of Inflammation, Oswaldo Cruz Institute, Oswaldo Cruz Foundation (Fiocruz), Rio de Janeiro, Brazil; ^2^ 2National Institute of Science and Technology on Neuroimmunomodulation (INCT-NIM), Oswaldo Cruz Institute, Oswaldo Cruz Foundation (Fiocruz), Rio de Janeiro, Brazil

**Keywords:** angiotensin II receptors, diabetes, glucocorticoids, renin-angiotensin system, steroidogenesis

## Abstract

Prior investigation shows an increase in the activity of both hypothalamus-pituitary-adrenal (HPA) axis and the renin-angiotensin system (RAS) in diabetic patients. Moreover, activation of angiotensin-II type 1 receptor (AT_1_) has been associated with adrenal steroidogenesis. This study investigates the role of RAS on the overproduction of corticosterone in diabetic mice. Diabetes was induced by intravenous injection of alloxan into fasted Swiss-webster mice. Captopril (angiotensin-converting enzyme inhibitor), Olmesartan (AT_1_ receptor antagonist), CGP42112A (AT_2_ receptor agonist) or PD123319 (AT_2_ receptor antagonist) were administered daily for 14 consecutive days, starting 7 days post-alloxan. Plasma corticosterone was evaluated by ELISA, while adrenal gland expressions of AT_1_ receptor, AT_2_ receptor, adrenocorticotropic hormone receptor MC2R, pro-steroidogenic enzymes steroidogenic acute regulatory protein (StAR), and 11β-hydroxysteroid dehydrogenase type 1 (11βHSD1) were assessed using immunohistochemistry or western blot. Diabetic mice showed adrenal gland overexpression of AT_1_ receptor, MC2R, StAR, and 11βHSD1 without altering AT_2_ receptor levels, all of which were sensitive to Captopril or Olmesartan treatment. In addition, PD123319 blocked the ability of Olmesartan to reduce plasma corticosterone levels in diabetic mice. Furthermore, CGP42112A significantly decreased circulating corticosterone levels in diabetic mice, without altering the overexpression of MC2R and StAR in the adrenal glands. Our findings revealed that inhibition of both angiotensin synthesis and AT_1_ receptor activity reduced the high production of corticosterone in diabetic mice *via* the reduction of MC2R signaling expression in the adrenal gland. Furthermore, the protective effect of Olmesartan on the overproduction of corticosterone by adrenals in diabetic mice depends on both AT_1_ receptor blockade and AT_2_ receptor activation.

## Introduction

Diabetes is a chronic metabolic disease characterized by a deficiency in insulin secretion, action, or both, followed by hyperglycemia (1). The reduction in circulating levels and/or in the activity of insulin culminates in a profound hormonal imbalance in diabetic patients, with an impact on several endocrine systems such as the hypothalamus-pituitary-adrenal (HPA) axis and the renin-angiotensin system (RAS) (2–4). Uncontrolled hyperglycemia in both diabetic patients and diabetes-induced (5) animals is associated with hyperactivity of the HPA axis (6), attested by high circulating levels of glucocorticoid hormones (7). Furthermore, treatment of diabetic animals with insulin normalized the systemic levels of glucocorticoids (3). We previously showed that the hyperactivity of HPA axis in diabetic animals was related to an increased expression of ACTH receptor MC2R in the adrenal glands (7). Several comorbidities of diabetes, including neuropathy, memory impairment, and wound healing deficiency (8), were correlated with the high levels of glucocorticoids in the circulation, mainly in poorly controlled blood glucose conditions.

As observed with the HPA axis, diabetic patients also show hyperactivity of the RAS with a marked increase in circulating levels of angiotensin (Ang) II (9, 10). Interestingly, the treatment of diabetic animals with Ang-II inhibitors improved neuropathy, memory impairment, and wound healing deficiency (11–13), suggesting that the RAS can regulate the HPA axis and *vice-versa* in diabetes. Ang-II is usually produced after the conversion of Ang-I by angiotensin-converting enzyme (ACE) (14) and can act through two Ang-II receptors (AT) subtypes AT_1_ and AT_2_ receptors (15). Ang-II has higher affinity for AT_1_ receptor, which promote vasoconstriction, fibrosis, and inflammation, than AT_2_ receptor, its contra-regulatory receptor that leads to anti-inflammatory, anti-fibrotic, and vasodilatory effects (16, 17). In addition, adrenal glands express both AT_1_ and AT_2_ receptors, and the blocked AT_1_ receptor inhibits the stress-induced release of glucocorticoids (17, 18). Furthermore, diabetic rats presented an increased expression of AT_1_ receptor in the adrenal gland (19).

Thus, once it has been observed that diabetic patients and animals present high circulating levels of Ang-II and the activation of AT_1_ receptor is involved in the stress-induced glucocorticoid production by adrenal glands, in this study, we evaluated the contribution of RAS to the overproduction of corticosterone in alloxan-diabetic mice.

## Material and methods

### Chemicals

Captopril, Olmesartan, CGP42112A, and alloxan monohydrate were purchased from Sigma Chemical Co. (Saint Louis, MO, USA). PD123319 from Cayman Chemical Co. (Ann Arbor, MI, USA). Ethanol, methanol, and xylene from Merck (Rio de Janeiro, RJ, Brazil). Sodium heparin and sterile saline solution from Roche (São Paulo, SP, Brazil). All solutions were prepared immediately before use.

### Animals and diabetes induction

Male Swiss-Webster mice (5-6 weeks old with 20 to 25g) were obtained from Oswaldo Cruz Foundation breeding colony and used in accordance with the guidelines of the Committee on Use of Laboratory Animals of Oswaldo Cruz Institute (CEUA-IOC/Fiocruz, license L-027/2016). Diabetes was randomly and blinded designated induced in 12 hours fasted mice (water *ad libitum*) by a single intravenous injection of alloxan monohydrate (65 mg/kg) (20) diluted with sterile saline (0.9% NaCl). Only mice showing two successive determinations of blood glucose levels (3 and 7 days after alloxan injection) greater than 16.7 mmol/L were considered diabetic and included in the experiment. Blood glycemia was determined with a glucose monitor (On Call Plus, San Diego, CA, USA) from samples obtained from the tail vein.

### Treatments

The animals were randomly designated into four experimental groups: non-diabetic untreated (n=6); non-diabetic treated with Captopril (n=6); diabetic untreated (n=6); diabetic treated with Captopril (n=6). In another set of experiments, the animals were randomly divided into four groups as follows: non-diabetic untreated (n=8); non-diabetic treated with Olmesartan (n=8); diabetic untreated (n=8); diabetic treated with Olmesartan (n=8). In the third set of experiments, the animals were randomly assigned to five groups as follows: non-diabetic untreated (n=6); diabetic untreated (n=6); diabetic treated with Olmesartan (n=6); diabetic treated with PD123319 (n=6); diabetic treated with Olmesartan plus PD123319 (n=6). In the last experiment setting, the animals were randomly assigned to four groups as follows: non-diabetic untreated (n=5); non-diabetic treated with CGP42112A (n=5); diabetic untreated (n=5); diabetic treated with CGP42112A (n=5).

Mice were treated with ACE inhibitor Captopril (10 mg/kg; gavage) (21), AT_1_ receptor antagonist Olmesartan (3 mg/kg; gavage) (22), AT_2_ receptor agonist CGP42112A (10 μg/kg; i.p.) (23), and/or AT_2_ receptor antagonist PD123319 (1 mg/kg; i.p.) (24) daily, during 14 consecutive days, starting 7 days after diabetes induction (**Figure S1**). Untreated mice received an equal volume of vehicle (sterile saline 0.9% NaCl) by gavage or i.p. in a final volume or gavage of 0.2 mL. All analyses were performed 24h after the last treatment. All samples were obtained and analyzed blindly.

### Hormone’s quantification

Mice were euthanized (ketamine 140 mg/Kg and xylazine 20 mg/Kg i.p.) during nadir (08:00h) of the circadian rhythm as described previously (25). Blood was immediately collected from the abdominal aorta with heparinized (40 U/ml) saline, centrifuged for 20 min at 1000 x *g* and stored at -20°C until use. Plasma corticosterone and Ang-II were detected by Elisa kit following the manufacture’s guidelines (Cayman Chemical, number 501320, Cedarlane Labs, Canada; Enzo Life Sciences, number ADI-900-204, NY, USA, respectively).

### Immunohistochemistry staining

The left adrenal glands were removed from mice and cleaned of surrounding fat. They were fixed in Millonig fixative solution for 24 h and embedded in paraffin. Then, sections of 3 μm were deparaffinized with xylene, rehydrated by a graded series of ethanol washes, and boiled in sodium citrate buffer (10 mM, pH 6.0) at the temperature of 95°C for 15 min to enhance antigen retrieval. Tissue sections were incubated with 3% H_2_O_2_ in methanol for 20 minutes to block endogenous peroxidases. To prevent non-specific binding, sections were incubated for 3 h with a solution containing 2% fetal bovine serum (FBS) and 8% goat serum dissolved in phosphate-buffered saline (PBS). Sections were incubated overnight with the specific antibody polyclonal rabbit anti-MC2R (1:50; Invitrogen ThermoFisher, Waltham, MA, USA) and anti-11βHSD1 (1:100; Santa Cruz Biotechnology, Santa Cruz, CA, USA) diluted with PBS, 2% FBS and 8% goat serum. Then, the adrenal slices were rinsed with PBS and incubated with the secondary antibodies horseradish peroxidase-conjugated streptavidin (HRP) (polyclonal anti-rabbit IgG (1:1.000), Invitrogen ThermoFisher System, MA, USA) for 2 h 30 min at room temperature followed by a 20-min exposure to the enzyme-substrate 3-amino-9-ethyl carbazole (AEC). The sections were counterstained using hematoxylin for 10 seconds to visualize the structure of the adrenals. As negative controls, the primary antibody was omitted. The slides were scanned using a 3DHISTECH–Panoramic MIDI whole slide scanner (Budapest, Hungary) and captured with a 20× objective lens. The analyses were performed using Image-Pro-Plus^®^ software version 6.2 (Media Cybernetics Inc, Bethesda, MA, USA), and the number of positive pixels was divided by the field area and expressed as pixels/μm^2^. The description of all antibodies used is in **Table S1**.

### Western blotting analysis

The cleaned right adrenal glands were homogenized in RIPA buffer containing protease and phosphatase inhibitor cocktails for western blotting analysis. After quantifying protein content by BCA method (26), 60 μg total protein/lane was resolved on 14% sodium dodecyl sulfate-polyacrylamide gel electrophoresis, and afterward electrotransferred through a semi-dry transfer apparatus (Trans-Blot SD; Bio-Rad, Hercules, CA, USA) to a nitrocellulose membrane. Then, the membrane was incubated for 90 minutes at 4°C in a blocking solution (Tris-buffered saline containing 5% bovine serum albumin and 0.1% Tween 20, pH 7.4 (TBST)) and, subsequently, incubated with a primary antibody dissolved in the blocking solution overnight at 4°C. Primary antibodies against the following proteins were used: anti-StAR (1:250; Santa Cruz Biotechnology, CA, USA), anti-AT_1_ receptor (1:250; Santa Cruz Biotechnology), anti-AT_2_ receptor (1:250; Santa Cruz Biotechnology), anti-MC2R (1:200; Santa Cruz Biotechnology), and anti-11βHSD1 (1:200; Santa Cruz Biotechnology). The housekeeping anti-β-actin (1:1000; Santa Cruz Biotechnology) was used as the standard. After incubation with an HRP conjugated secondary antibody polyclonal anti-rabbit IgG HRP (1:10.000, Invitrogen ThermoFisher System, MA, USA), polyclonal anti-goat or monoclonal anti-mouse IgGs HRP (1:1000, R&D System, Minneapolis, MN, USA) for 60 minutes at room temperature, the membranes were washed, and the immunocomplexes were visualized by using a chemiluminescence detection system on X-ray films (Kodak; PerkinElmer) or ChemiDoc MP Imaging System 6.0.1 (Bio-Rad Laboratories, Inc, Hercules, CA, USA). Then, the band density measurements were analyzed by Image J software 1.53a (Wayne Rasband, National Institutes of Health, USA) or Image Lab software version 6.1.0 (Bio-Rad Laboratories, Inc). The description of all antibodies used is in **Table S1**.

### Statistical analysis

The data are reported as mean ± standard error of mean (S.E.M.). All data were evaluated to ensure normal distribution and statistically analyzed by one-way ANOVA followed by Bonferroni *post hoc* test, with GraphPad Prism software version 8.0.1 (La Jolla, CA, USA). Probability values (P) of 0.05 or less were considered significant.

## Results

### ACE inhibition reduces the exacerbated adrenal glucocorticoid steroidogenesis observed in diabetic mice

**Figure 1:**
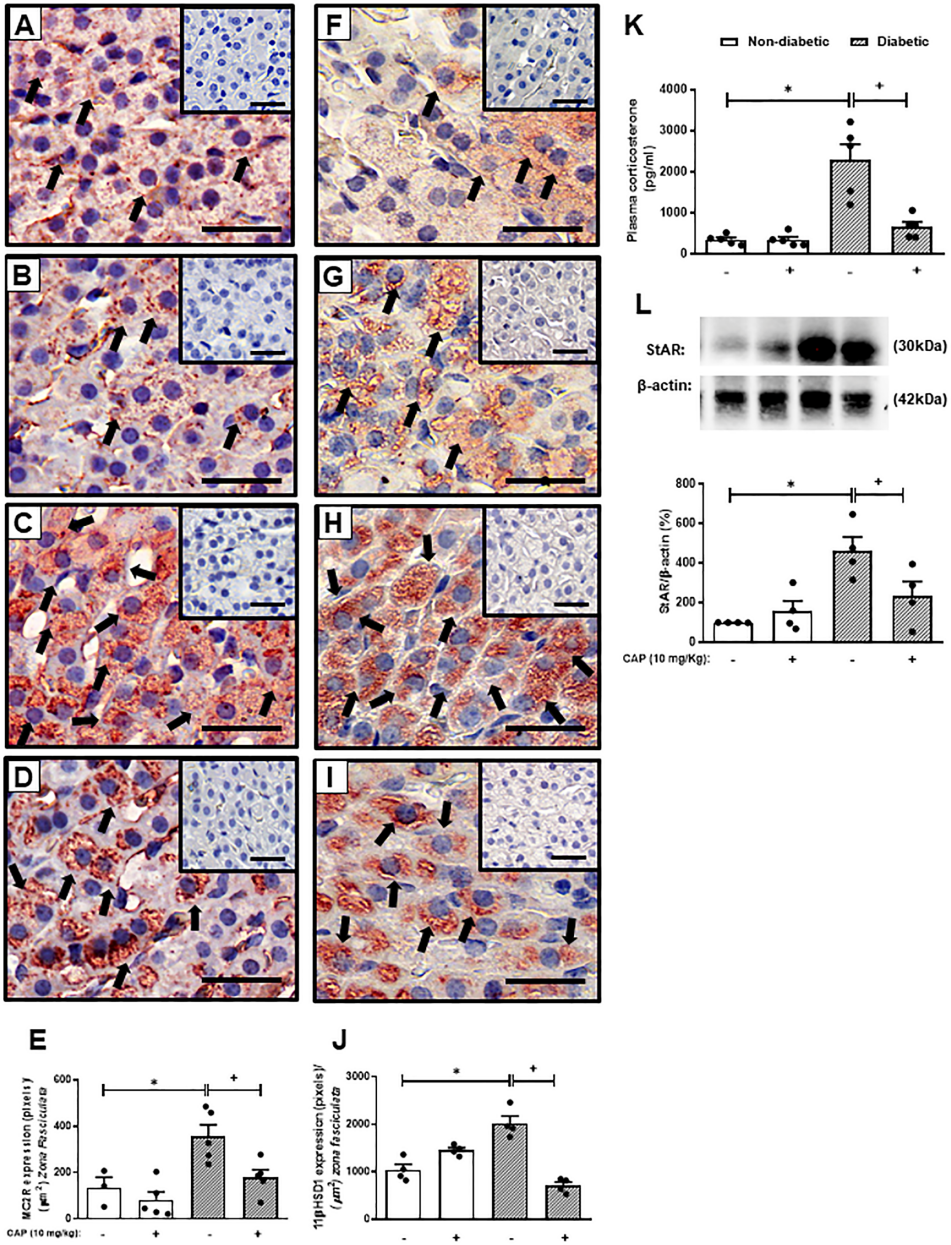
Captopril decreases steroidogenic machinery in the adrenal glands and plasma corticosterone levels in diabetic mice. Seven days after diabetes induction, Captopril (10 mg/kg, gavage) was given once a day for 14 consecutive days. Untreated animals received an equal amount of vehicle (0.9% sterile saline, gavage). The analyses were performed 21 days after diabetes induction. Panels show representative photomicrographs of the expression of MC2R in the *zona fasciculata* of adrenals of non-diabetic untreated **(A)**, non-diabetic treated with Captopril **(B)**, diabetic untreated **(C)**, and diabetic treated with Captopril mice **(D)**. Panels show representative photomicrographs of the expression of 11βHSD1 in the *zona fasciculata* of adrenals of non-diabetic untreated **(F)**, non-diabetic treated with Captopril **(G)**, diabetic untreated **(H)**, and diabetic treated with Captopril mice **(I)**. **(E, J)** Quantification of pixels associated with positive MC2R and 11βHSD1 expression, respectively. Inserts represent negative controls. Black arrows indicate immunolabelling of MC2R **(A-D)** and 11βHSD1 **(F-I)** in the *zona fasciculata* of adrenals. **(K)** Plasma quantification of corticosterone levels. **(L)** Expression of StAR in adrenal glands was determined by western blot. The data were normalized to β-actin and represented as the ratio between the expressions of StAR: β-actin relative to the control. Each value represents the mean ± S.E.M. ^*^ *P* < 0.05 compared to non-diabetic untreated mice. ^+^ *P* < 0.05 compared to diabetic untreated mice. Bar scale = 80 µm. Cap, Captopril.

As expected, hyperglycemia and loss of body weight confirmed the induction of diabetes with alloxan. We also showed that diabetes-induced an increase in the circulating levels of Ang-II as compared to non-diabetic mice. Treatment with Captopril significantly reduced the plasma Ang-II levels, confirming its inhibitory activity on ACE. Nevertheless, it did not alter body weight or circulating glucose levels in diabetic mice (**Table 1**). Immunohistochemistry evaluation of the adrenals of non-diabetic and diabetic mice revealed increased expression of ACTH receptor MC2R (**Figures 1A, C**, respectively) and steroidogenic enzyme 11βHSD1 (**Figures 1F, H**, respectively) in the condition of diabetes. Treatment with Captopril reduced the overexpression of both MC2R (**Figure 1D**) and 11βHSD1 (**Figure 1I**) in the adrenal glands of diabetic mice. However, it did not alter the expression of these proteins in non-diabetic mice (**Figures 1B, G**, respectively). Quantitative analyses of immunohistochemistry showed that Captopril significantly inhibited the expression of MC2R (**Figure 1E**) and 11βHSD1 (**Figure 1J**) in diabetic mice. We also showed that diabetes caused an increase of steroidogenic enzyme StAR (**Figures 1L** and **S2**) in the adrenal glands, in parallel to a rise in the plasma corticosterone levels (**Figure 1K**), compared to the non-diabetic ones (**Figures 1K, L**, respectively). In addition, the overexpression of StAR and the hypercorticoidism noted in diabetic mice were sensitive to treatment with Captopril, whereas ACE inhibitor did not alter these outputs in non-diabetic mice.

**Table 1:** Captopril reduces plasma levels of angiotensin II but does not alter body weight and hyperglycemia in alloxan-induced diabetic mice.

Group	Body Weight (g)	Glycaemia (mmol/L)	Plasma Ang-II (pg/ml)
**Non-diabetic**	42.21 ± 5.25	2.94 ± 0.355	7.16 ± 4.74
**Non-diabetic treated**	40.11 ± 3.32	3.69 ± 0.876	5.78 ± 2.27
**Diabetic**	31.82 ± 4.91*	23.44 ± 3.28*	16.50 ± 5.56*
**Diabetic treated**	28.08 ± 4.43	19.75 ± 6.021	8.77 ± 3.31**^+^**

Diabetes was induced by a single intravenous injection of alloxan (65 mg/kg), and the analyses were performed 21 days after diabetes induction. Values represent the mean ± S.E.M. * P <0.05 compared to non-diabetic mice. **+** P < 0.05 compared to non-treated diabetic mice.

### AT_1_ receptor blocking decreases the exacerbated adrenal glucocorticoid steroidogenesis observed in diabetic mice

**Figure 2:**
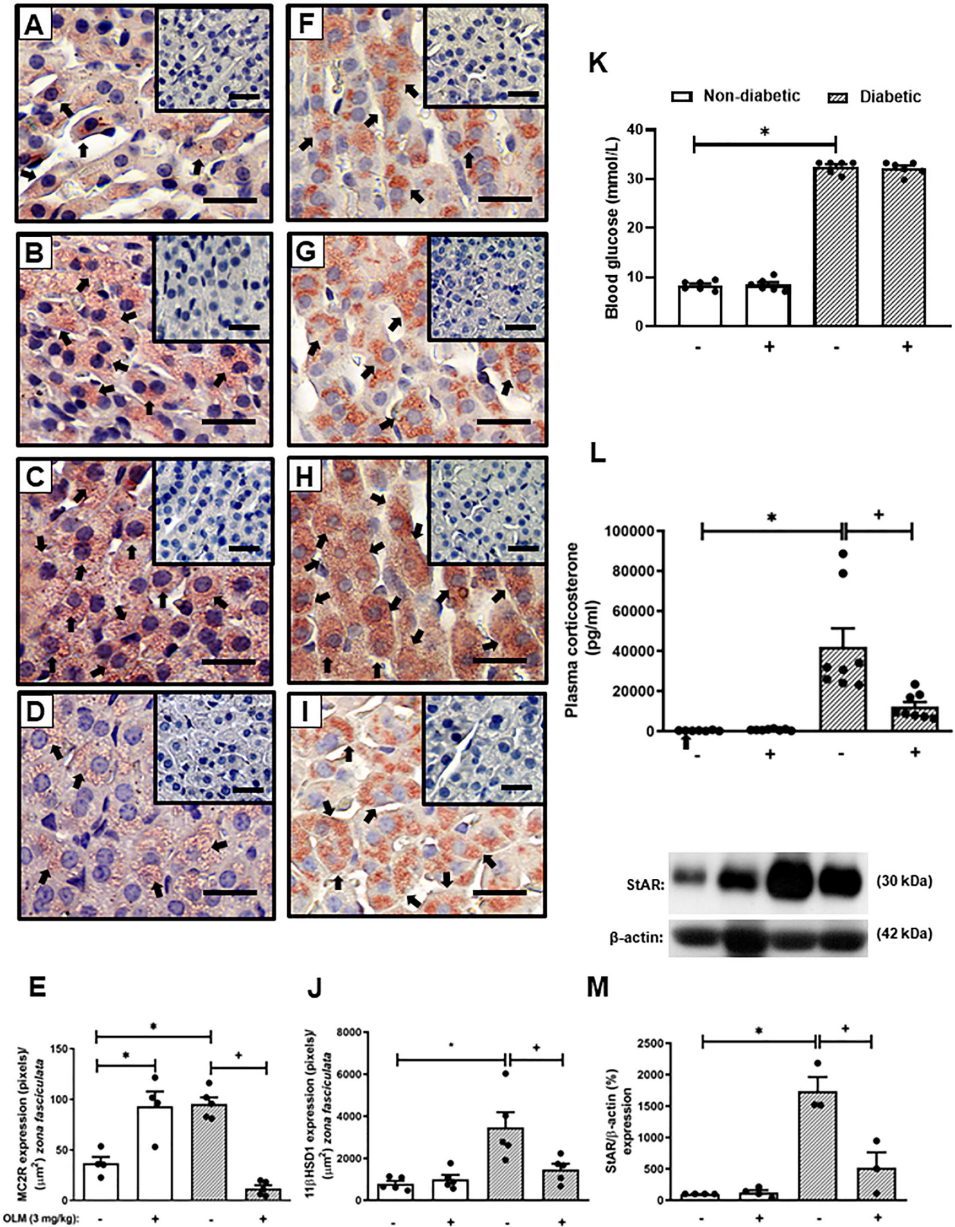
Olmesartan reduces steroidogenic machinery in the adrenal glands and plasma corticosterone levels in diabetic mice. Seven days after diabetes induction, Olmesartan (3 mg/kg, gavage) was given once a day for 14 consecutive days. Untreated animals received an equal amount of vehicle (0.9% sterile saline, gavage). The analyses were performed 21 days after diabetes induction. Panels show representative photomicrographs of the expression of MC2R in the *zona fasciculata* of adrenals of non-diabetic untreated **(A)**, non-diabetic treated with Olmesartan **(B)**, diabetic untreated **(C)**, and diabetic treated with Olmesartan mice **(D)**. Panels show representative photomicrographs of the expression of 11βHSD1 in the *zona fasciculata* of adrenals of non-diabetic untreated **(F)**, non-diabetic treated with Olmesartan **(G)**, diabetic untreated **(H)**, and diabetic treated with Olmesartan mice **(I)**. **(E, J)** Quantification of pixels associated with positive MC2R and 11βHSD1 expression, respectively. Inserts represent negative controls. Black arrows indicate immunolabelling of MC2R **(A-D)** and 11βHSD1 **(F-I)** in the *zona fasciculata* of adrenals. **(K)** Blood glucose quantification. **(L)** Plasma quantification of corticosterone levels. **(M)** Expression of StAR in adrenal glands was determined by western blot. The data were normalized to β-actin and represented as the ratio between the expressions of StAR: β-actin relative to the control. Each value represents the mean ± S.E.M. ^*^ *P* < 0.05 compared to non-diabetic untreated mice. ^+^ *P* < 0.05 compared to diabetic untreated mice. Bar scale = 80 µm. Olm, Olmesartan.

The reversion of adrenal steroidogenesis increase shown in diabetic mice by Captopril suggests that activation of AT_1_ receptor by Ang-II is involved in the hypercorticoidism noted in these animals. To prove this hypothesis, we treated the animals with an AT_1_ receptor antagonist. Treatment with Olmesartan did not modify systemic glucose levels in both non-diabetic and diabetic mice (**Figure 2K**). Nevertheless, immunohistochemistry evaluation of the adrenal of diabetic mice treated with Olmesartan showed reduced expression of MC2R (**Figure 2D**) and 11βHSD1 (**Figure 2I**) as compared to untreated diabetic mice (**Figures 2C, H**, respectively). Treatment with Olmesartan increased the expression of MC2R (**Figure 2B**). Still, it did not alter 11βHSD1 (**Figure 2G**) in the adrenal glands of non-diabetic mice compared to untreated non-diabetic ones (**Figures 2A, F**, respectively). Quantitative analyses of immunohistochemistry showed that Olmesartan significantly inhibited the expression of MC2R (**Figure 2E**) and 11βHSD1 (**Figure 2J**) in diabetic mice. Treatment with Olmesartan significantly reduced both StAR expression (**Figures 2M** and **S3**) in adrenal glands and the high levels of plasma corticosterone (**Figure 2L**) in diabetic mice without modifying these outputs in non-diabetic mice.

### Captopril and Olmesartan inhibit the overexpression of AT_1_ receptor in the adrenal glands of diabetic mice

**Figure 3:**
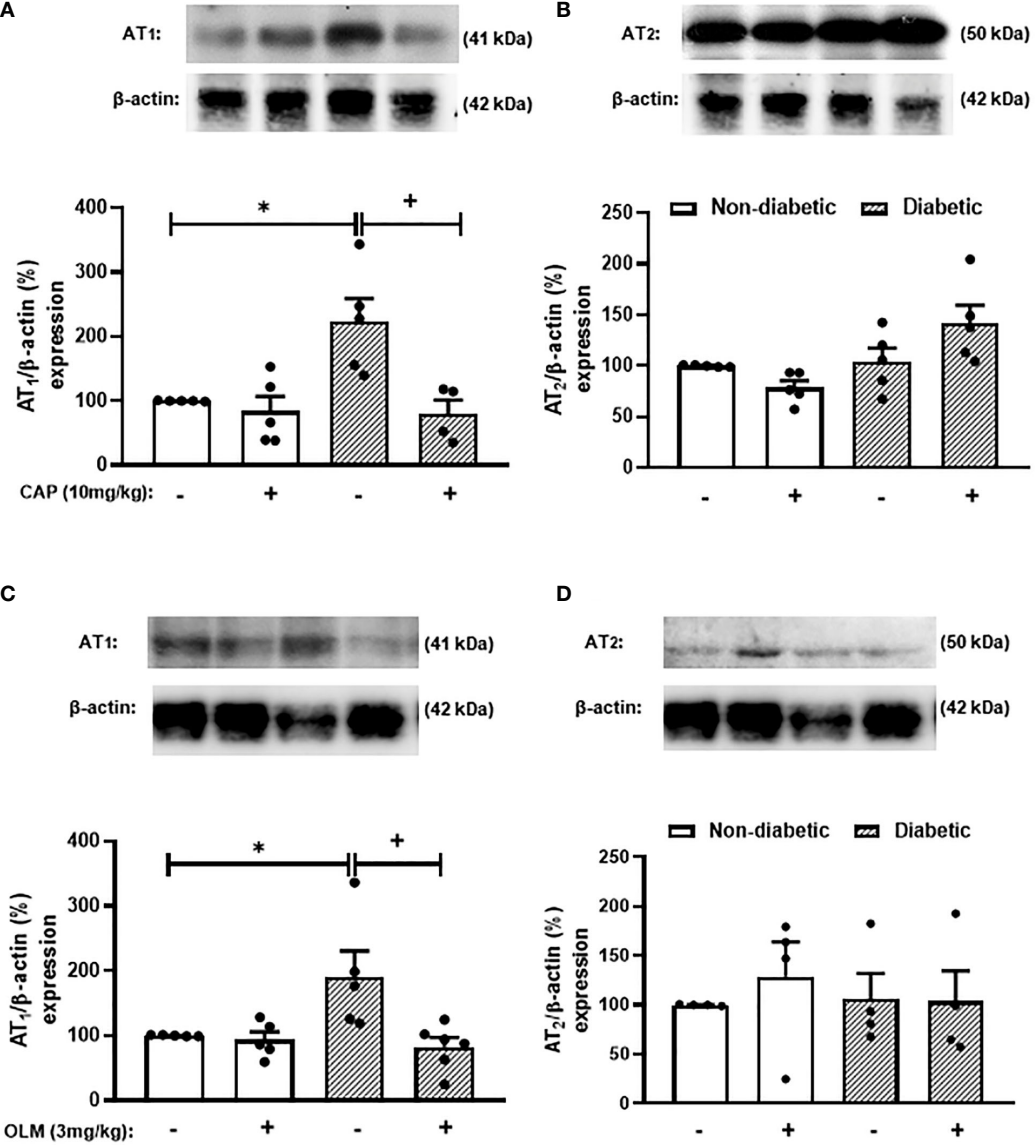
Captopril and Olmesartan decrease the overexpression of AT_1_ receptor in the adrenals of diabetic mice. Seven days after diabetes induction, Captopril (10 mg/kg, gavage) **(A, B)** or Olmesartan (3 mg/kg, gavage) **(C, D)** was given once a day for 14 consecutive days. Untreated animals received an equal amount of vehicle (0.9% sterile saline, gavage). The analyses were performed 21 days after diabetes induction. Expression of AT_1_ **(A, C)** and AT_2_ receptors **(B, D)** in adrenal glands from diabetic mice was determined by western blot. The data were normalized to β-actin and represented as the ratio between the expressions of AT_1_: β-actin and AT_2_: β-actin relative to the control. Each value represents the mean ± S.E.M. ^*^ *P* < 0.05 compared to non-diabetic untreated mice. ^+^ *P* < 0.05 compared to diabetic untreated mice. Cap, Captopril; Olm, Olmesartan.

While trying to clarify the mechanism underlying the Ang-II-induced exacerbation of adrenal glucocorticoid steroidogenesis in diabetic mice, we evaluated the expression of Ang-II receptors in the adrenal gland of mice. We showed that diabetes provoked an increase in the expression of AT_1_ receptor in the adrenals of mice compared to non-diabetic mice (**Figures 3A, C**, **S4**, and **S5**). However, diabetes did not interfere with AT_2_ receptor expression in this gland (**Figures 3B, D**, **S4**, and **S5**). The expression of AT_1_ receptor in the adrenal glands of diabetic mice was sensitive to treatments with both Captopril (**Figures 3A** and **S4**) and Olmesartan (**Figures 3C** and **S5**). In addition, both treatments did not modify the expression of AT_2_ receptor in the adrenal glands of diabetic mice (**Figures 3B, D**, **S4**, and **S5**).

### Activation of AT_2_ receptor participates in the reduction of circulating corticosterone levels induced by Olmesartan

**Figure 4:**
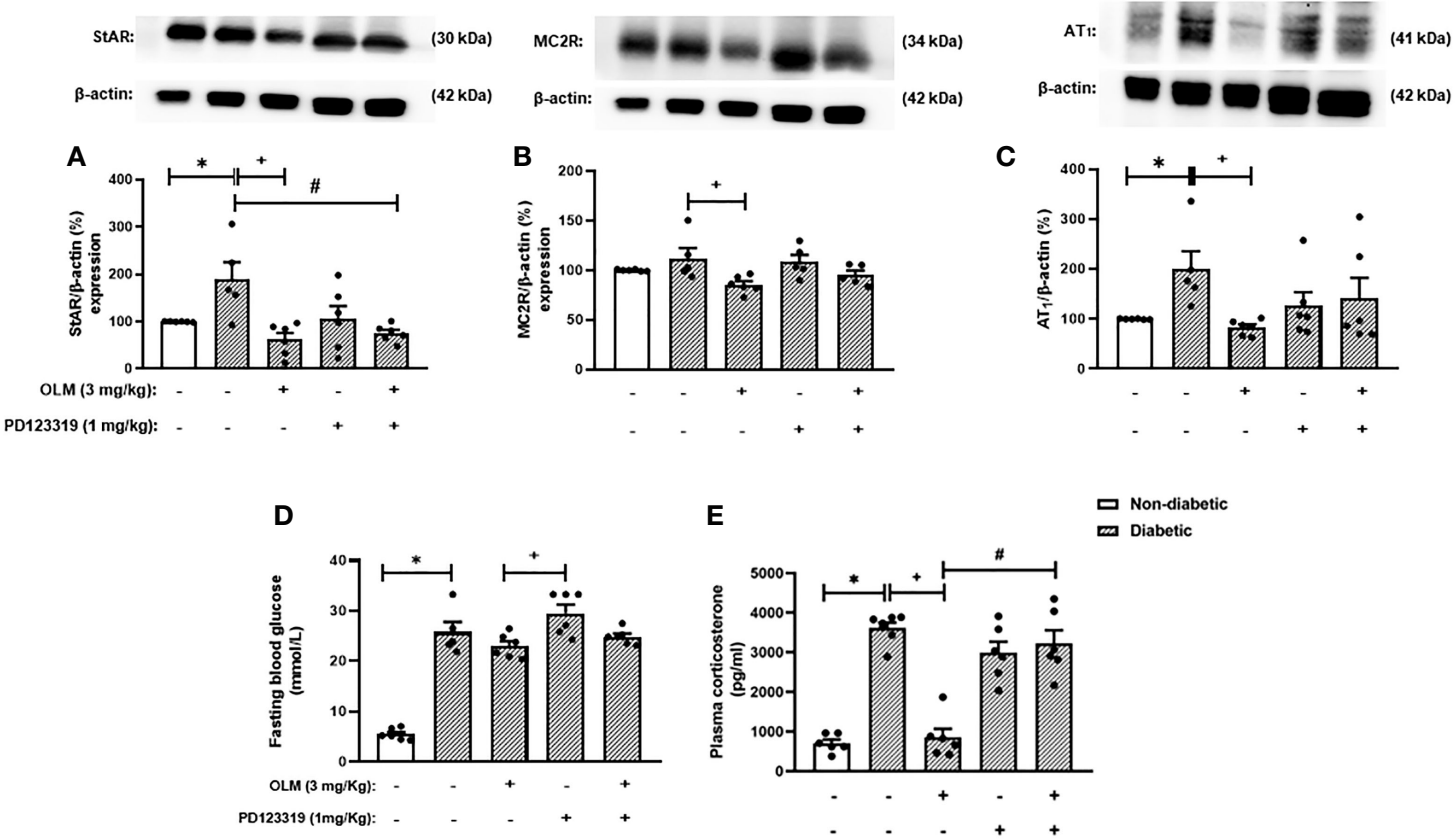
Blockade of AT_2_ receptor impaired Olmesartan mediated decrease of plasma corticosterone levels in diabetic mice. Seven days after diabetes induction, Olmesartan (3 mg/kg, gavage) and PD123319 (1 mg/kg, i.p.) were given once a day for 14 consecutive days. Untreated animals received an equal amount of vehicle (0.9% sterile saline, gavage). The analyses were performed 21 days after diabetes induction. **(A-C)** Expression of StAR, MC2R, and AT_1_ receptor in the adrenal glands, respectively, was determined by western blot. The data were normalized to β-actin and represented as the ratio between the expressions of StAR: β-actin, MC2R: β-actin, and AT_1_: β-actin relative to the control. **(D)** Blood glucose quantification. **(E)** Plasma quantification of corticosterone levels. Each value represents the mean ± S.E.M. ^*^ *P* < 0.05 compared to non-diabetic untreated mice. ^+^ *P* < 0.05 compared to diabetic untreated mice. ^#^ *P* < 0.05 compared to diabetic mice treated with Olmesartan. Olm, Olmesartan; PD, PD123319.

Since the blockage of AT_1_ receptor culminates in the activation of AT_2_ receptor by Ang-II and its receptor reduces inflammation, we hypothesized that this pathway could be involved with the inhibitory effect of Olmesartan on the exacerbated adrenal steroidogenesis observed in diabetic mice. We treated diabetic mice concomitantly with both AT_1_ and AT_2_ receptors antagonists to attest to this theory. We showed that either Olmesartan, AT_2_ receptor antagonist PD123319, or Olmesartan plus PD123319 did not alter systemic glucose levels in non-diabetic and diabetic mice (**Figure 4D**). We also confirmed that Olmesartan significantly decreased the high levels of plasma corticosterone in diabetic mice compared to untreated diabetic mice. However, the treatment with AT_2_ receptor antagonist PD123319 did not alter the hypercorticoidism observed in diabetic mice. Interestingly, treatment with PD123319 blocked the inhibitory properties of Olmesartan on the rise of systemic corticosterone levels in diabetic mice (**Figure 4E**), suggesting that the effect of AT_1_ receptor blockage involves the activation of AT_2_ receptor by Ang-II. Nevertheless, the treatment with PD123319 did not modify the Olmesartan-induced reduction in the expression of StAR (**Figures 4A** and **S6**), MC2R (**Figures 4B** and **S6**), and AT_1_ receptor (**Figures 4C** and **S6**) in the adrenal glands of diabetic mice. In addition, these outputs were not changed by treatment with PD123319 in diabetic mice.

### Activation of AT_2_ receptor decreases the exacerbated adrenal glucocorticoid steroidogenesis observed in diabetic mice

**Figure 5:**
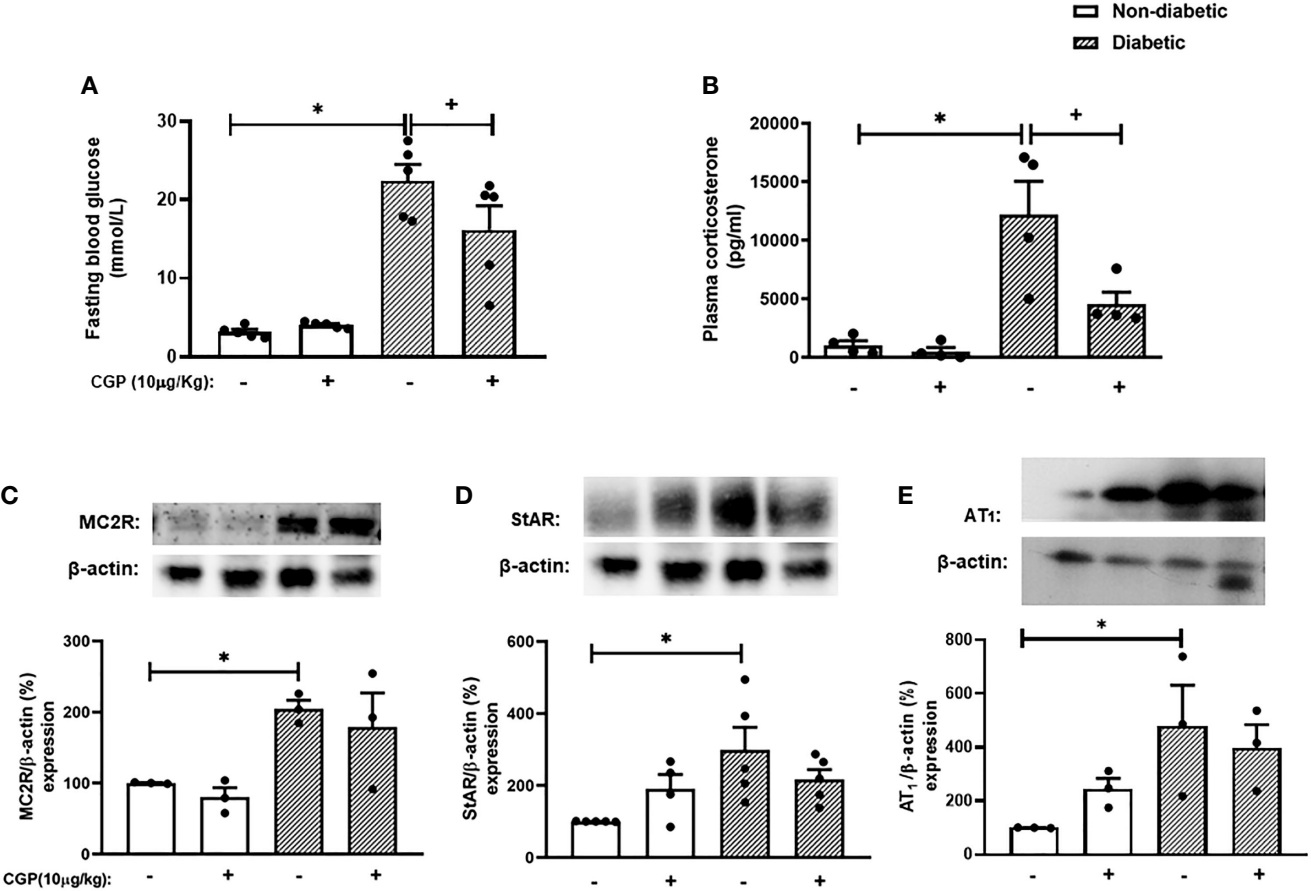
CGP42112A reduces plasma corticosterone levels but did not modify AT_1_, MC2R or StAR expression in the adrenal glands of diabetic mice. Seven days after diabetes induction, CGP42112A (10 µg/kg, i.p.) was given once a day for 14 consecutive days. Untreated animals received an equal amount of vehicle (0.9% sterile saline, gavage). The analyses were performed 21 days after diabetes induction. **(A-C)** Expression of StAR, MC2R, and AT_1_ receptor in the adrenal glands, respectively, was determined by western blot. The data were normalized to β-actin and represented as the ratio between the expressions of StAR: β-actin, MC2R: β-actin, and AT_1_: β-actin relative to the control. **(D)** Blood glucose quantification. **(E)** Plasma quantification of corticosterone levels. Each value represents the mean ± S.E.M. ^*^ *P* < 0.05 compared to non-diabetic untreated mice. ^+^ *P* < 0.05 compared to diabetic untreated mice. CGP, CGP42112A.

To confirm whether activation of AT_2_ receptor can inhibit the exacerbation of adrenal steroidogenesis in diabetic mice, we treated the animals with the AT_2_ receptor agonist CGP42112A. Interestingly, CGP42112A significantly decreased blood glucose levels. Nevertheless, diabetic mice treated with CGP42112A remained hyperglycemic, but had substantially lower blood glucose levels than those of untreated diabetic mice. In addition, treatment with CGP42112A did not modify systemic glucose levels in non-diabetic mice (**Figure 5A**). Finally, we showed that the rise of corticosterone levels (**Figure 5B**) observed in diabetic mice was sensitive to the treatment with CGP42112A. However, it did not modify the expression of MC2R (**Figures 5C** and **S7**), StAR (**Figures 5D** and **S7**), and AT_1_ receptor (**Figures 5E** and **S8**) in the adrenal glands of diabetic mice. In addition, these outputs were not changed by treatment with CGP42112A in non-diabetic mice.

## Discussion

This study provides new perspectives on the effect of Ang-II on the exacerbation of adrenal glucocorticoid steroidogenesis in diabetes. We showed that the inhibition of Ang-II synthesis and the blockade of AT_1_ receptor, using Captopril and Olmesartan, respectively, reduced plasma corticosterone levels in diabetic mice *via* downregulation of MC2R expression in the adrenal gland. Diabetic mice presented an overexpression of AT_1_ receptor in the adrenal glands without altering the content of AT_2_ receptor. The treatments with either Captopril or Olmesartan decreased the expression of AT_1_ receptor in the adrenal gland of diabetic mice. Furthermore, the Olmesartan-induced reduction of adrenal glucocorticoid steroidogenesis observed in diabetic mice was reversed by treatment with the AT_2_ receptor antagonist PD123319. In addition, the treatment of diabetic mice with AT_2_ receptor agonist CGP42112A reduced plasma corticosterone levels. Our findings indicate that the high circulating levels of Ang-II in diabetic mice could account for the exacerbation of corticosterone production by adrenal glands through the activation of AT_1_ receptor. In addition, the AT_1_ receptor antagonist Olmesartan-induced reduction of corticosterone levels observed in diabetic mice is related to the activation of AT_2_ receptor.

In diabetes, the high production of Ang-II and activation of AT_1_ receptor promote oxidative stress generation and systemic inflammation (16). In this work, we used Captopril as an ACE inhibitor to investigate the impact of Ang-II on the exacerbation of adrenal glucocorticoid steroidogenesis in diabetic mice. First, we noted that Captopril inhibited the increased levels of Ang-II in the plasma of diabetic mice, indicating that the treatment effectively inhibited ACE in our model. We previously demonstrated that alloxan-induced diabetes increased circulating corticosterone levels in parallel to augmentation in the MC2R expression in the adrenal glands (7). Here, we confirmed these data and showed that diabetic mice also presented an increase in the expression of both steroidogenic enzymes StAR and 11βHSD1 in the adrenal gland. Treatment with Captopril decreased all these outcomes in diabetic mice without modifying the hyperglycemia. Our data strongly suggest that the overproduction of Ang-II is involved with the exacerbation of corticosterone production by adrenal glands in diabetic mice. The Captopril-induced reduction in the StAR and 11βHSD1 in the adrenal gland of diabetic mice is in line with previous data that showed an up-regulation of both steroidogenic enzymes after activation of the Ang-II/AT_1_ receptor pathway (27, 28).

To confirm whether the Ang-II/AT_1_ receptor pathway participates in the high corticosterone production by adrenal glands of diabetic mice, we treated the animals with the AT_1_ receptor antagonist Olmesartan. We showed that treatment with Olmesartan reduced the expression of MC2R, StAR, and 11βHSD1 in the adrenal gland of diabetic mice and decreased the systemic levels of corticosterone. The inhibition seen with the treatment of diabetic mice with Olmesartan was very similar to that observed when using Captopril, suggesting that the exacerbation of adrenal glucocorticoid steroidogenesis in diabetic mice is related to the activation of the Ang-II/AT_1_ receptor pathway. These data agree with others that demonstrated that the blocking of AT_1_ receptor inhibited the stress-induced release of glucocorticoids (18). Next, we evaluated whether the effect of Captopril and Olmesartan could be related to an alteration in the expression of Ang-II receptors in the adrenal gland of diabetic mice. We showed that diabetic mice presented an increase in the expression of AT_1_ receptor in the adrenal gland, but it did not alter the content of AT_2_ receptor. These data agree with the literature that shows an increase in the expression of AT_1_ receptor in the adrenal gland of diabetic rats (19). The treatments with either Captopril or Olmesartan significantly reduced the expression of AT_1_ receptor in the adrenal gland of diabetic mice but did not modify the AT_2_ receptor density. These findings reinforce our hypothesis that corticosterone-producing cells in the adrenal glands of diabetic mice are probably more responsive to Ang-II stimuli since the adrenals of these mice overexpress AT_1_ receptor (29). Besides, the upregulation of AT_1_ receptor expression seems to be a positive loop dependent on the Ang-II/AT_1_ receptor pathway activation, confirmed by the sensitivity to Captopril and Olmesartan treatments.

Since the activation of the Ang-II/AT_2_ receptor system showed opposite effects to the Ang-II/AT_1_ receptor pathway regarding inflammation, proliferation, and fibrosis (28), we evaluated whether the activation of its receptor participates in the Olmesartan inhibitory effect on plasma corticosterone levels of diabetic mice. For this, we treated the animals concomitantly with both AT_1_ and AT_2_ receptors antagonists, Olmesartan and PD123319, respectively. We used this approach because the Ang-II formed by diabetic animals will bind to AT_2_ receptor with the AT_1_ receptor inhibition (30, 31). We observed that the ability of Olmesartan to reduce diabetes-induced hypercorticoidism was blocked with the co-treatment with PD123319. These data suggested that treatment with Olmesartan reduced corticosterone levels in diabetic mice by inhibiting AT_1_ receptor and, consequently, providing a greater activation of AT_2_ receptor in the adrenals by Ang-II. Nevertheless, PD123319 did not reverse the Olmesartan-induced reduction in the expression of MC2R, StAR, and AT_1_ receptors in the adrenal glands, suggesting that anti-steroidogenic effect of AT_2_ receptor activation does not depend on this molecular pathway.

To prove the hypothesis that the activation of AT_2_ receptor can decrease the hypercorticoidism observed in diabetic mice, we treated the animals with the AT_2_ receptor agonist CGP42112A. We showed that treatment with CGP42112A reduced the systemic levels of corticosterone in diabetic mice, but it did not alter the expression of MC2R, StAR, and AT_1_ receptor in the adrenal gland. These data confirm that activation of AT_2_ receptor is essential to the reduction of glucocorticoid levels observed with the treatment of diabetic mice with AT_1_ receptor antagonist Olmesartan. Nevertheless, the mechanism by which the CGP42112A reduced steroidogenesis in diabetic mice is distinct from the blockage of AT_1_ receptor since it did not modify the expression of the ACTH and AT_1_ receptors or steroidogenic enzyme StAR in the adrenals. Contrary to what we showed, AT_2_ receptor agonist reduced the overexpression of AT1 receptor in the hypothalamus of deoxycorticosterone acetate/NaCl-induced hypertension rats (32). These data strongly suggest that the mechanism by which activation of AT_2_ receptor reduces corticosterone production by adrenals of diabetic mice is different from that associated with the hyperproduction of this hormone induced by AT_1_ receptor stimulation. On the other hand, treatment with CGP42112A significantly reduced glycemia in diabetic mice, which could explain, at least in part, the reduction in the production of corticosterone by the adrenals (33). Although CGP42112A, a peptide AT_2_ receptor agonist, possesses less stability compared to non-peptide AT_2_ receptor agonist compound 21 (C21), both agonists show significant antioxidant, anti-inflammatory, anti-fibrotic, anti-hypertrophic, and anti-apoptotic properties. Furthermore, different from our findings with CGP42112A, studies have shown that treatment with C21 in streptozotocin-induced diabetic animals did not change blood glucose or HbA1c (34–36). In diabetic mice, the Ang-1-7 cascade might be stimulated by the AT_2_ receptor pathway through ACE2, once ACE2 has a potential role in alterations in glucose tolerance and insulin secretion (37, 38).

One limitation of our study is that in alloxan-induced diabetes the β-pancreatic cell death is not induced by an autoimmune response, as observed in type 1 diabetic patients (39). Nevertheless, this murine model of diabetes shows several clinical illnesses noted in patients, including hyperglycemia, hypoinsulinemia, retinopathy, neuropathy, and impaired wound healing (40). Consistently, in the alloxan-induced diabetes model, animals presented hypercorticoidism and increased Ang-II circulating levels, as detected in patients with type 1 diabetes (7, 9, 10). In addition, although the effects of ACE inhibition are usually accredited to reduce Ang-II formation, it is well known that this pharmacological approach provokes an increase in the circulating levels of Ang-1-7 (41). Therefore, an unanswered question is whether Captopril-induced inhibition of plasma corticosterone levels in diabetic mice is only related to the Ang-II production drop. This question arises since Ang-1-7 has contra-regulatory effects on AT_1_ receptor activation, as observed with the stimulation of the Ang-II/AT_2_ receptor pathway (42). Another limitation of our study is the absence of experiments using tissue-specific knockout animals for both AT_1_ and AT_2_ receptors in the adrenals to confirm if our results depend on local or systemic actions of the agonists and/or antagonists of these Ang-II receptors.

In summary, our results indicate that inhibition of Ang-II synthesis by Captopril and blockade of AT_1_ receptor by Olmesartan reduced the exacerbation of adrenal glucocorticoid steroidogenesis in diabetic mice by down-regulation of the expression of ACTH receptor and steroidogenic enzymes StAR and 11βHSD1 in the adrenal gland. Furthermore, activation of AT_2_ receptor also seems to be important to the Olmesartan-induced decrease of adrenal glucocorticoid steroidogenesis in diabetic mice. With the data obtained in this study, we believe that ACE inhibitors, AT_1_ receptor blockers, or AT_2_ receptor agonists will become an essential target for treating type 1 diabetes and other diseases associated with hypercorticoidism in the future.

## Data availability statement

The original contributions presented in the study are included in the article/**Supplementary Material**. Further inquiries can be directed to the corresponding author.

## Ethics statement

The animal study was reviewed and approved by committee on Use of Laboratory Animals of the Oswaldo Cruz Institute (CEUA-IOC/FIOCRUZ, license L-027/2016).

## Author contributions

ASC contributed to conception and design of the study, performed experiments, analyzed 28 the data, and wrote the manuscript. NSM and DBRI performed experiments and analyzed the data. PMRS and MAM analyzed the data, contributed with essential reagents or tools and to preparation of the manuscript. VFC contributed to conception and design of the study, with essential reagents or tools, analyzed the data, and wrote the manuscript. All authors contributed to the article and approved the submitted version.

## Funding

This study was financial supported by Fundação Carlos Chagas de Amparo à Pesquisa do Estado do Rio de Janeiro (FAPERJ), Conselho Nacional de Desenvolvimento Cientıífico e Tecnoloígico (CNPq), Programa de Auxıílio à Pesquisa (PAPESVI/FIOCRUZ), Programa INOVA FIOCRUZ, and Ministeírio da Sauíde, Brazil. The funding agencies had no role in the study design, data collection and analysis, decision to publish or preparation of the manuscript.

## Acknowledgments

We express our gratitude to Raissa D. Ventura from FIOCRUZ for her technical support.

## Conflict of interest

The authors declare that the research was conducted in the absence of any commercial or financial relationships that could be construed as a potential conflict of interest.

## Publisher’s note

All claims expressed in this article are solely those of the authors and do not necessarily represent those of their affiliated organizations, or those of the publisher, the editors and the reviewers. Any product that may be evaluated in this article, or claim that may be made by its manufacturer, is not guaranteed or endorsed by the publisher.

## Abbreviations

11βHSD1, 11β-hydroxysteroid dehydrogenase type 1; ACE, Angiotensin-converting enzyme; ACE2, Angiotensin-converting enzyme-2; ACTH, Adrenocorticotrophic hormone; AEC, 3-amino-9-ethyl carbazole; Ang-II, Angiotensin II; Ang-1-7, angiotensin 1-7; AT1, Angiotensin II type 1 receptor; AT2, Angiotensin II type 2 receptor; FBS, fetal bovine serum; HPA, Hypothalamus-pituitary-adrenal; HRP, horseradish peroxidase-conjugated streptavidin; HbA1c, Glycated hemoglobin A1c; MC2R, Melanocortin 2 receptor; PBS, phosphate-buffered saline; RAS, Renin-angiotensin system; StAR - Steroidogenic acute regulatory protein; TBST, Tris-buffered saline containing 5% bovine serum albumin and 0.1% Tween 20.

## References

[1] Alam U Asghar O Azmi S Malik RA. General aspects of diabetes mellitus. Handbook of Clinical Neurology. Diabetes and the Nervous System. 1st ed. Elsevier B.V (2014). 126:211–21. doi: 10.1016/B978-0-444-53480-4.00015-1 25410224

[2] Diz-Chaves Y Gil-Lozano M Toba L Fandiño J Ogando H González-Matías LC. Stressing diabetes? the hidden links between insulinotropic peptides and the HPA axis. J Endocrinol (2016) 230:R77–94. doi: 10.1530/JOE-16-0118 27325244

[3] Chan O Inouye K Akirav E Park E Riddell MC Vranic M. Insulin alone increases hypothalamo-pituitary-adrenal activity, and diabetes lowers peak stress responses. Endocrinology (2005) 146:1382–90. doi: 10.1210/en.2004-0607 15564337

[4] Clark KA Jacob EB MohanKumar PS MohanKumar SMJ. Leptin and HPA axis activity in diabetic rats: Effects of adrenergic agonists. Brain Res (2019) 1707:54–61. doi: 10.1016/j.brainres.2018.11.025 30468724 PMC13321493

[5] Lenzen S. The mechanisms of alloxan- and streptozotocin-induced diabetes. Diabetologia (2008) 51:216–26. doi: 10.1007/s00125-007-0886-7 18087688

[6] Trifunovic S Stevanovic I Milosevic A Ristic N Janjic M Bjelobaba I. The function of the hypothalamic–Pituitary–Adrenal axis during experimental autoimmune encephalomyelitis: Involvement of oxidative stress mediators. Frontiers in Neuroscience (2021) 15. doi: 10.3389/fnins.2021.649485 PMC8248369 34220419

[7] Torres RC Magalhães NS Silva PMRE Martins MA Carvalho VF. Activation of PPAR-γ reduces HPA axis activity in diabetic rats by up-regulating PI3K expression. Exp Mol Pathol (2016) 101:290–301. doi: 10.1016/j.yexmp.2016.10.002 27725163

[8] Torres RC Prevatto JP Silva PMR Martins MA Carvalho VF. From type-1 diabetes HPA axis to the disease complications. J Diabetes Metab (2013) S12:002. doi: 10.4172/2155-6156.S12-002

[9] Rahimi Z Rahimi Z Moradi M Nasri H. A systematic review of the role of renin angiotensin aldosterone system genes in diabetes mellitus, diabetic retinopathy, and diabetic neuropathy. J Res Med Sci (2014) 19 (11):1090–8. PMC4310085 25657757

[10] Senanayake Pd Bonilha VL W Peterson J Yamada Y Karnik SS Daneshgari F. Retinal angiotensin II and angiotensin-(1-7) response to hyperglycemia and an intervention with captopril. J Renin-Angiotensin-Aldosterone System (2018) 19(3):1–11. doi: 10.1177/1470320318789323 PMC6104213 30126320

[11] Coppey LJ Davidson EP Rinehart TW Gellett JS Oltman CL Lund DD. ACE inhibitor or angiotensin II receptor antagonist attenuates diabetic neuropathy in streptozotocin-induced diabetic rats. Diabetes (2006) 55(2):341–8. doi: 10.2337/diabetes.55.02.06.db05-0885 16443766

[12] Kamber M Papalazarou V Rouni G Papageorgopoulou E Papalois A Kostourou V. Angiotensin II inhibitor facilitates epidermal wound regeneration in diabetic mice. Front Physiol (2015) 6:170. doi: 10.3389/fphys.2015.00170 26106332 PMC4460301

[13] Lai M-C Wu S-N Huang C-W. Telmisartan, an antagonist of angiotensin II receptors, accentuates voltage-gated na+ currents and hippocampal neuronal excitability. Front Neurosci (2020) 14:902. doi: 10.3389/fnins.2020.00902 33013297 PMC7499822

[14] Daimon M Kamba A Murakami H Takahashi K Otaka H Makita K. Association between pituitary-adrenal axis dominance over the renin-angiotensin-aldosterone system and hypertension. J Clin Endocrinol Metab (2016) 101:889–97. doi: 10.1210/jc.2015-3568 26731257

[15] Chow BSM Allen TJ. Angiotensin II type 2 receptor (AT2R) in renal and cardiovascular disease. Clin Sci (2016) 130:1307–26. doi: 10.1042/CS20160243 27358027

[16] Bernardi S Michelli A Zuolo G Candido R Fabris B. Update on RAAS modulation for the treatment of diabetic cardiovascular disease. J Diabetes Res (2016) 2016:1–17. doi: 10.1155/2016/8917578 PMC5019930 27652272

[17] Ames MK Atkins CE Pitt B. The renin-angiotensin-aldosterone system and its suppression. J Vet Intern Med (2019) 33:363–82. doi: 10.1111/jvim.15454 PMC6430926 30806496

[18] Daimon M Kamba A Murakami H Mizushiri S Osonoi S Matsuki K. Dominance of the hypothalamus-pituitary-adrenal axis over the renin-angiotensin-aldosterone system is a risk factor for decreased insulin secretion. Sci Rep (2017) 7:1–9. doi: 10.1038/s41598-017-10815-y 28900121 PMC5596009

[19] Mansour MH Al-Qattan K Thomson M Ali M. Garlic (Allium sativum) down-regulates the expression of angiotensin II AT1 receptor in adrenal and renal tissues of streptozotocin-induced diabetic rats. Inflammopharmacology (2013) 21:147–59. doi: 10.1007/s10787-012-0139-3 22644380

[20] Insuela DBR Ferrero MR Gonçalves-de-Albuquerque CF Chaves A da S da Silva AYO Castro-Faria-Neto HC. Glucagon reduces neutrophil migration and increases susceptibility to sepsis in diabetic mice. Front Immunol (2021) 12:633540. doi: 10.3389/fimmu.2021.633540 34295325 PMC8290340

[21] Diao TY Pan H Gu SS Chen X Zhang FY Wong MS. Effects of angiotensin-converting enzyme inhibitor, captopril, on bone of mice with streptozotocin-induced type 1 diabetes. J Bone Miner Metab (2014) 32:261–70. doi: 10.1007/s00774-013-0500-7 23934056

[22] Yamashita C Hayashi T Mori T Matsumoto C Kitada K Miyamura M. Efficacy of olmesartan and nifedipine on recurrent hypoxia-induced left ventricular remodeling in diabetic mice. Life Sci (2010) 86:322–30. doi: 10.1016/j.lfs.2009.12.013 20060397

[23] Kljajic ST Widdop RE Vinh A Welungoda I Bosnyak S Jones ES. Direct AT2 receptor stimulation is athero-protective and stabilizes plaque in apolipoprotein e-deficient mice. Int J Cardiol (2013) 169:281–7. doi: 10.1016/j.ijcard.2013.09.015 24161533

[24] Chun-ye M Yin L. Neuroprotective effect of angiotensin II type 2 receptor during cerebral ischemia/reperfusion. Neural Regeneration Research (2016) 11(7):1102–7. doi: 10.4103/1673-5374.187044 PMC4994452 27630693

[25] Ventura RD Chaves AS Magalhães NS Gonzalez FB Pacini MF Pérez AR. Activation of PPARγ reduces n-acetyl-cysteine -induced hypercorticoidism by down-regulating MC2R expression into adrenal glands. Free Radic Biol Med (2020) 156:137–43. doi: 10.1016/j.freeradbiomed.2020.06.008 32574682

[26] Paula L D’almeida A Pacheco De Oliveira MT de Souza ÉT de Sá Coutinho D Ciambarella BT Gomes CR. α-bisabolol-loaded lipid-core nanocapsules reduce lipopolysaccharide-induced pulmonary inflammation in mice. Int J Nanomed (2017) 12:4479–91. doi: 10.2147/IJN.S130798 PMC5484570 28684908

[27] Ayari H Legedz L Cerutti C Lantelme P Feugier P Gustin MP. Mutual amplification of corticosteroids and angiotensin systems in human vascular smooth muscle cells and carotid atheroma. J Mol Med (2014) 92:1201–8. doi: 10.1007/s00109-014-1193-7 25088215

[28] Ghorayeb N Bourdeau I Lacroix A. Role of ACTH and other hormones in the regulation of aldosterone production in primary aldosteronism. Front Endocrinol (Lausanne) (2016) 7:72. doi: 10.3389/fendo.2016.00072 27445975 PMC4921457

[29] Zhang S Morrison JL Gill A Rattanatray L MacLaughlin SM Kleemann D. Dietary restriction in the periconceptional period in normal-weight or obese ewes results in increased abundance of angiotensin-converting enzyme (ACE) and angiotensin type 1 receptor (AT1R) in the absence of changes in ACE or AT1R methylation in the adre. Reproduction (2013) 146:443–54. doi: 10.1530/REP-13-0219 24084173

[30] Zhou Z Peters AM Wang S Janda A Chen J Zhou P. Reversal of aortic enlargement induced by increased biomechanical forces requires AT1R inhibition in conjunction with AT2R activation. Arterioscler Thromb Vasc Biol (2019) 39:459–66. doi: 10.1161/ATVBAHA.118.312158 PMC6400319 30602301

[31] Liles C Li H Veitla V Liles JT Murphy TA Cunningham MW. AT2R autoantibodies block angiotensin II and AT1R autoantibody-induced vasoconstriction. Hypertension (2015) 66:830–5. doi: 10.1161/HYPERTENSIONAHA.115.05428 PMC4567423 26259590

[32] Dai S Zhang Y Peng W Shen Y He J. Central infusion of angiotensin II type 2 receptor agonist compound 21 attenuates DOCA/NaCl-induced hypertension in female rats. Oxid Med Cell Longev (2016) 2016:3981790. doi: 10.1155/2016/3981790 26783414 PMC4691472

[33] Taguchi K Matsumoto T Kamata K Kobayashi T. Angiotensin II type 2 receptor-dependent increase in nitric oxide synthase activity in the endothelium of db/db mice is mediated *via* a MEK pathway. Pharmacol Res (2012) 66:41–50. doi: 10.1016/J.PHRS.2012.02.010 22465219

[34] Matavelli LC Zatz R Siragy HM. A nonpeptide angiotensin II type 2 receptor agonist prevents renal inflammation in early diabetes. J Cardiovasc Pharmacol (2015) 65:371–6. doi: 10.1097/FJC.0000000000000207 PMC4390440 25590749

[35] Koulis C Bryna SMC McKelvey M Ulrike MS Unger T Thallas-Bonke V. AT2R 75 agonist, Compound 21, Is reno-protective against type 1 diabetic nephropathy. Hypertension (2015) 65:1073–81. doi: 10.1161/HYPERTENSIONAHA.115.05204 25776077

[36] Pandey A Gaikwad AB. AT2 receptor agonist compound 21: A silver lining for diabetic nephropathy. Eur J Pharmacol (2017) 815:251–7. doi: 10.1016/j.ejphar.2017.09.036 28943106

[37] Batlle D Soler MJ Ye M. ACE2 and diabetes: ACE of ACEs? Diabetes (2010) 59:2994. doi: 10.2337/DB10-1205 21115782 PMC2992757

[38] Paulis L Foulquier S Namsolleck P Recarti C Muscha Steckelings U Unger T. Combined angiotensin receptor modulation in the management of cardio-metabolic disorders. Drugs (2016) 76:1–12. doi: 10.1007/s40265-015-0509-4 26631237 PMC4700059

[39] Quan N Ho E Lai W Tsai Y-H Bray T. Administration of NF-kappaB decoy inhibits pancreatic activation of NF-kappaB and prevents diabetogenesis by alloxan in mice. FASEB J (2001) 15:1616–8. doi: 10.1096/FJ.00-0855FJE 11427504

[40] King AJF. The use of animal models in diabetes research. Br J Pharmacol (2012) 166:877–94. doi: 10.1111/J.1476-5381.2012.01911.X PMC3417415 22352879

[41] Giani JF Gironacci MM Muñoz MC Peña C Turyn D Dominici FP. Angiotensin-(1-7) stimulates the phosphorylation of JAK2, IRS-1 and akt in rat heart *in vivo*: Role of the AT1 and mas receptors. Am J Physiol Heart Circ Physiol (2007) 293:1154–63. doi: 10.1152/ajpheart.01395.2006 17496209

[42] Muñoz MC Burghi V Miquet JG Cervino IA Quiroga DT Mazziotta L. Chronic blockade of the AT2 receptor with PD123319 impairs insulin signaling in C57BL/6 mice. Peptides (NY) (2017) 88:37–45. doi: 10.1016/j.peptides.2016.12.003 27979738

