# Supplementary material for: Effect of the renin-angiotensin system on the exacerbation of adrenal glucocorticoid steroidogenesis in diabetic mice: Role of angiotensin-II type 2 receptor
Source: Front Endocrinol (Lausanne). 2022 Nov 17;13:1040040. doi: 10.3389/fendo.2022.1040040 (PMC9712183; doi:10.3389/fendo.2022.1040040)
Supplement: Supplementary file 1 [file DataSheet_1.docx]

**Effect of the renin-angiotensin system on the exacerbation of adrenal glucocorticoid steroidogenesis in diabetic mice: role of angiotensin-II type 2 receptor**

Amanda S. Chaves^1^, Nathalia S. Magalhães^1^, Daniella B.R. Insuela^1^, Patrícia M. R. e Silva^1^, Marco A. Martins^1^, and Vinicius F. Carvalho^1,2^*.

^1^Laboratório de Inflamação, Instituto Oswaldo Cruz, Fundação Oswaldo Cruz; Av. Brasil, nº 4365, Manguinhos, CEP 21045-900, Rio de Janeiro, Brazil.

^2^Instituto Nacional de Ciência e Tecnologia em Neuroimunomodulação (INCT-NIM).

Supplementary Material

**Methods (SM)**

**Animals**

Mice were housed in microisolator cage type II with wood shavings in groups of 2-4 in a temperature- humidity- and light-controlled (12 h light: 12 h darkness cycle) colony room. The animals were also maintained in environmental enrichment, including shredded paper, plastic tubes, and mouse Igloo, throughout the experiment. The microisolator cages, chow diet, and drinking bottles, with a total capacity of 250 mL of water, were autoclaved to eliminate possible contamination with pathogens.

We assessed mice’s welfare through observation of piloerection, locomotion, feces alteration, water consumption, and amount of urine. Diabetic animals show an increased in water consumption and urine excretion rate, so we provided new water and exchanged animal cages for clean ones every day. Nonetheless, no alterations in piloerection, locomotion, and feces alterations were observed in our animals.

# Supplementary Figures and Tables

## Supplementary Tables

**Table S1: Description of antibodies used in the immunohistochemistry and western blot**

| Antibodies | Dilution Range | Trademark | Analysis | MW | Isotype |
| --- | --- | --- | --- | --- | --- |
| Anti-MC2R (PA5-25454) | 1:50 | Invitrogen ThermoFisher | IHQ | 34 kDa | IgG |
| Anti-11βHSD1 (H-100) | 1:100 | Santa Cruz | IHQ | 33 kDa | IgG |
| Anti-rabbit (31460) | 1:1000 | Invitrogen ThermoFisher | IHQ | - | IgG |
| Anti-StAR (D-2) | 1:250 | Santa Cruz | Western Blot | 30 kDa | IgG |
| Anti-At1 (N-10) | 1:250 | Santa Cruz | Western Blot | 41 kDa | IgG |
| Anti-At2 (H-143) | 1:250 | Santa Cruz | Western Blot | 50 kDa | IgG |
| Anti-MC2R (H-70) | 1:200 | Santa Cruz | Western Blot | 34 kDa | IgG |
| Anti-beta actin (C4) | 1:1000 | Santa Cruz | Western Blot | 42 kDa | IgG |
| Anti-beta actin (D6A8) | 1:1000 | Cell Signaling | Western Blot | 42 kDa | IgG |
| Anti-rabbit H+L (31460) | 1:10.000 | Invitrogen ThermoFisher | Western Blot | - | IgG |
| Anti-mouse HRP (HAF007) | 1:1000 | R&D | Western Blot | - | IgG |
| Anti-rabbit HRP (HAF008) | 1:1000 | R&D | Western Blot | - | IgG |

## Supplementary Figures


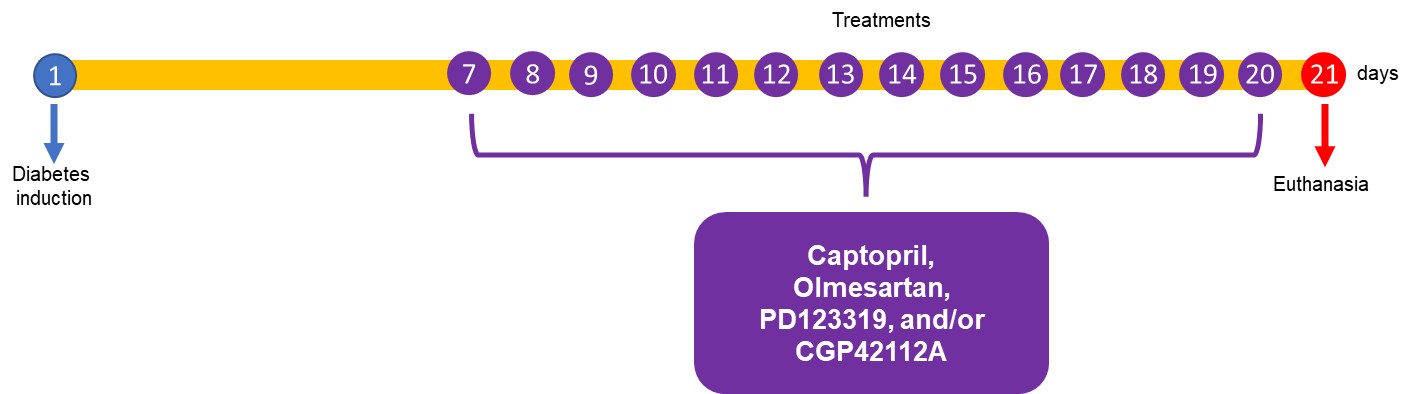


**Figure S1.** Schematic overview of study protocols for induction of diabetes and treatments in mice. The animals were treated with Captopril (10 mg/kg; gavage), Olmesartan (3 mg/kg; gavage), PD123319 (1 mg/kg; i.p.), and/or CGP42112A (10 μg/kg; i.p.) daily, during 14 consecutive days, starting 7 days after diabetes induction.

A

B


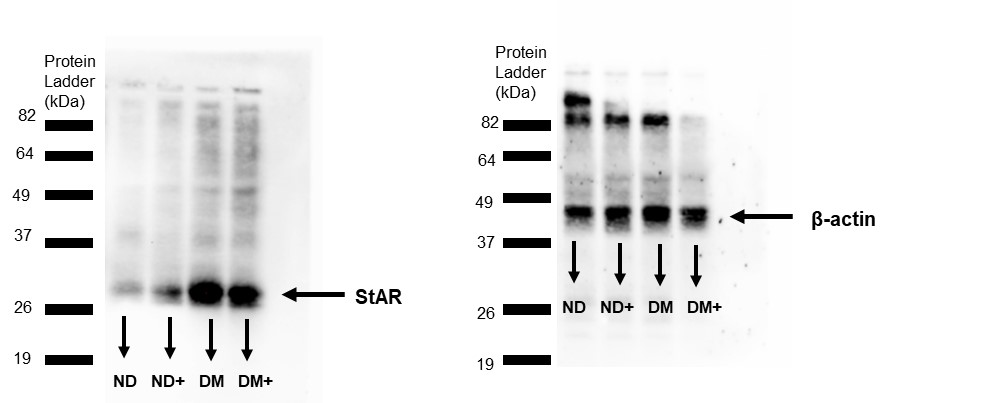


**Figure S2:** **Western blot evaluation of StAR expression in adrenal glands of diabetic mice treated with Captopril.** Representative full-length blots of StAR **(A)** and β-actin **(B)**. Analysis of StAR and β-actin expression were performed by western blot. All antibodies were validated by the producers and experiments. ND = non-diabetic mice; ND+ = non-diabetic mice treated with Captopril; DM = diabetic mice, and DM+ = diabetic mice treated with Captopril.

B

A


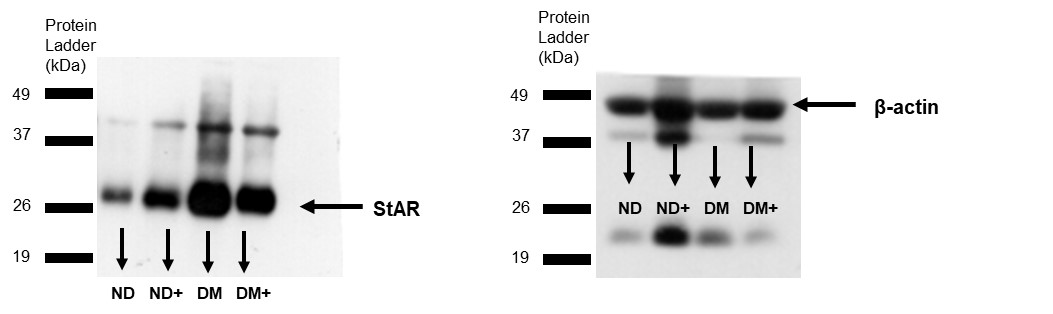


**Figure S3:** **Western blot evaluation of StAR expression in adrenal glands of diabetic mice treated with Olmesartan.** Representative full-length blots of StAR **(A)** and β-actin **(B)**. Analysis of StAR and β-actin expression were performed by western blot. All antibodies were validated by the producers and experiments. ND = non-diabetic mice; ND+ = non-diabetic mice treated with Olmesartan; DM = diabetic mice, and DM+ = diabetic mice treated with Olmesartan.

C

B

A


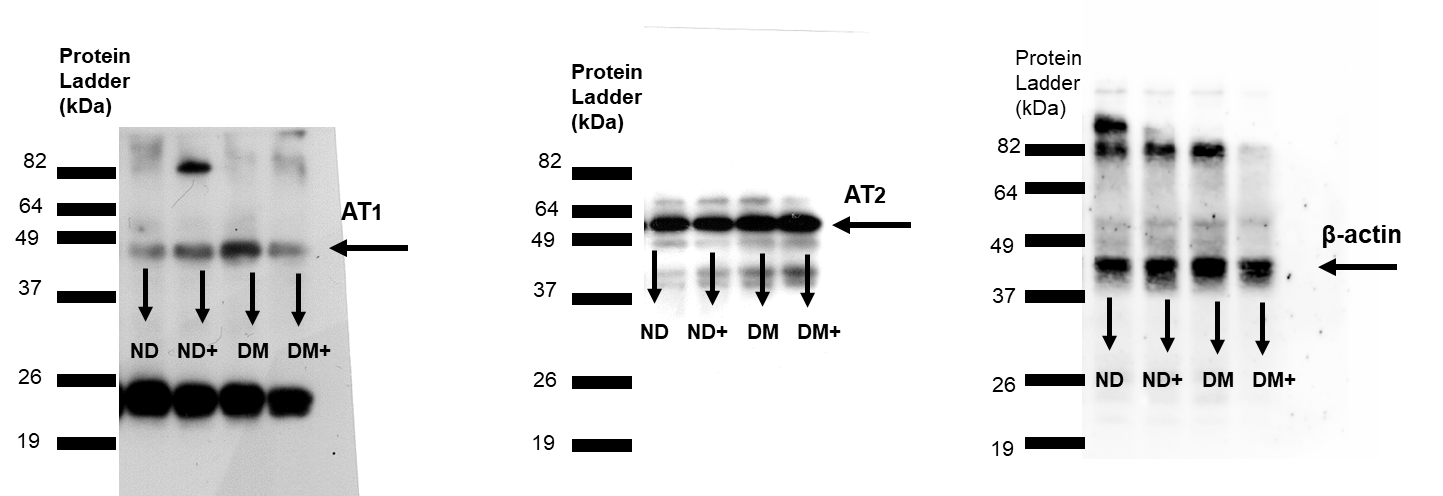


**Figure S4:** **Western blot evaluation of AT_1_ and AT_2_ receptors expression in adrenal glands of diabetic mice treated with Captopril.** Representative full-length blots of AT_1_ receptor **(A)**, AT_2_ receptor **(B)**, and β-actin **(C)**. Analysis of AT_1_, AT_2_ and β-actin expression were performed by western blot. All antibodies were validated by the producers and experiments. ND = non-diabetic mice; ND+ = non-diabetic mice treated with Captopril; DM = diabetic mice, and DM+ = diabetic mice treated with Captopril.

B

A

C


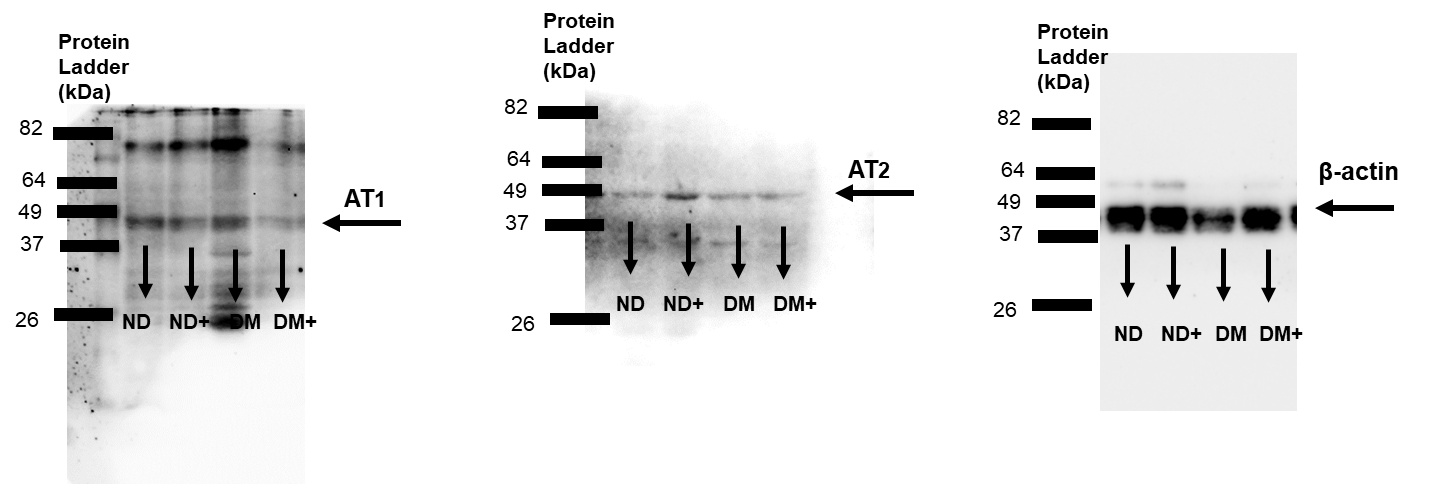


**Figure S5:** **Western blot evaluation of AT_1_ and AT_2_ expression in adrenal glands of diabetic mice treated with Olmesartan.** Representative full-length blots of AT_1_ **(A)**, AT_2_ **(B)**, and β-actin **(C)**. Analysis of AT_1_, AT_2_ and β-actin expression were performed by western blot. All antibodies were validated by the producers and experiments. ND = non-diabetic mice; ND+ = non-diabetic mice treated with Olmesartan; DM = diabetic mice, and DM+ = diabetic mice treated with Olmesartan.

D

C

B

A


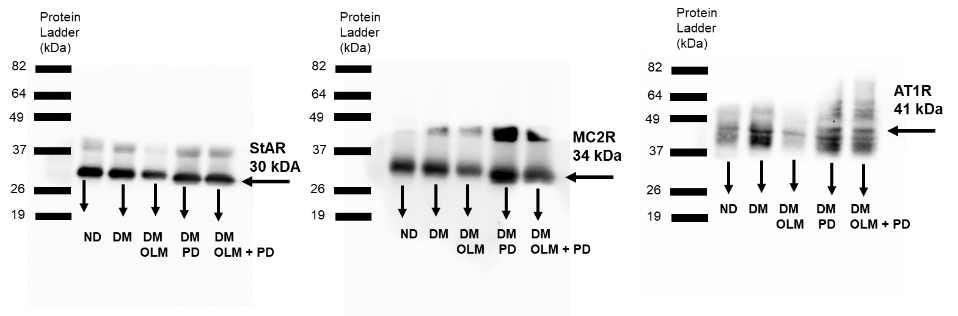

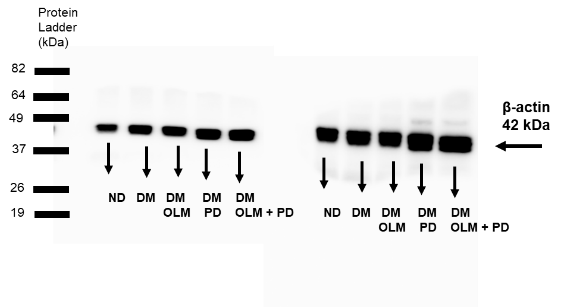


**Figure S6:** **Western blot evaluation of StAR, MC2R, and AT_1_ receptor expression in adrenal glands of diabetic mice treated with both Olmesartan and PD123319.** Representative full-length blots of StAR **(A)**, MC2R **(B),** AT_1_ **(C)** and β-actin **(D)**. Analysis of StAR, MC2R, AT_1_, and β-actin expression were performed by western blot. All antibodies were validated by the producers and experiments. ND = non-diabetic mice; DM = diabetic mice non-treated; DM+OLM = diabetic mice treated with OLM; DM+PD = diabetic mice treated with PD, and DM+OLM+PD = diabetic mice treated with OLM and PD. OLM = Olmesartan; PD= PD123319.

C

B

A


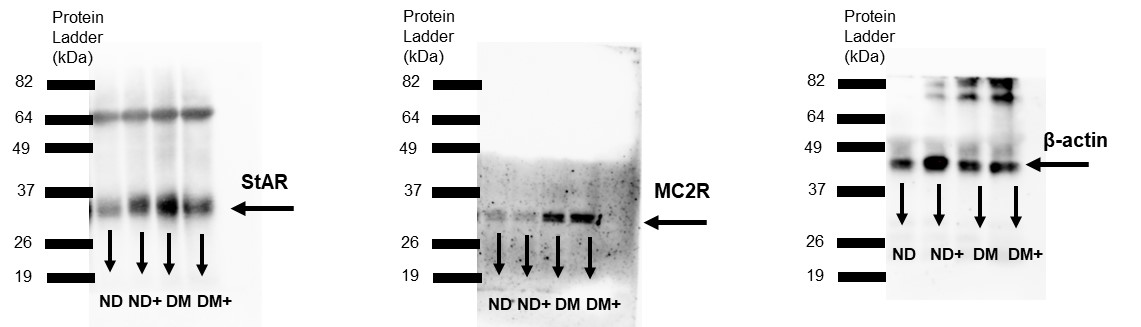


**Figure S7:** **Western blot evaluation of StAR and MC2R expression in adrenal glands of diabetic mice treated with CGP42112A.** Representative full-length blots of StAR **(A)**, MC2R **(B)** and β-actin **(C)**. Analysis of StAR, MC2R and β-actin expression were performed by western blot. All antibodies were validated by the producers and experiments. ND = non-diabetic mice; ND+ = non-diabetic mice treated with CGP42112A; DM = diabetic mice, and DM+ = diabetic mice treated with CGP42112A.

A

B


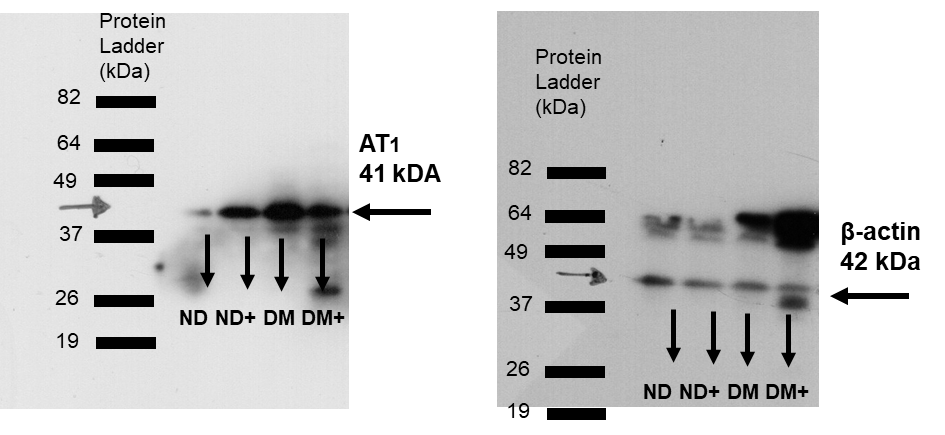


**Figure S8:** **Western blot evaluation of AT_1_ receptor expression in adrenal glands of diabetic mice treated with CGP42112A.** Representative full-length blots of AT_1_ **(A)** and β-actin **(B)**. Analysis of AT_1_ and β-actin expression were performed by western blot. All antibodies were validated by the producers and experiments. ND = non-diabetic mice; ND+ = non-diabetic mice treated with CGP42112A; DM = diabetic mice, and DM+ = diabetic mice treated with CGP42112A.
